# Enhanced biosorption of cadmium ions on immobilized surface-engineered yeast using cadmium-binding peptides

**DOI:** 10.3389/fmicb.2024.1496843

**Published:** 2024-11-15

**Authors:** Songting Wang, Yongmei Sun, Shihong Wang, Chunkun Fan, Daojie Wang, Fei Liu, Haiyan Zhang

**Affiliations:** ^1^School of Life Sciences, Henan University, Kaifeng, China; ^2^Engineering Research Center for Applied Microbiology of Henan Province, Kaifeng, China; ^3^Institute of Agriculture Research, Tibet Academy of Agricultural and Animal Husbandry Science, Tibet, China

**Keywords:** cadmium-binding peptide, phage peptide library, biosorption, surface-engineered yeast, immobilization

## Abstract

A new type of cadmium (Cd) ion cell surface adsorbent was developed by integrating bacteriophage display peptide library technology with cell surface display technology. Cd^2+^ chelating resin served as the target molecule in screening experiments, leading to the identification of four Cd^2+^ −binding peptides. These peptides were introduced into *Saccharomyces cerevisiae* via the pYD1 plasmid using lithium acetate heat shock transformation. Adsorption efficiency tests indicated that the engineered yeasts adsorbed more Cd^2+^ than the control strain EBY100 when exposed to the same amount of Cd^2+^. Among these peptides, sequence 3-containing strain was demonstrated to have the highest Cd^2+^ adsorption efficiency, being 35% higher than the control strain. Additionally, when this recombinant yeast strain was immobilized using sodium alginate, the adsorption efficiency was increased by 55.7% compared to the control strain.

## Introduction

Heavy metal pollution is a global environmental concern (Meena et al., 2018; Rahman and Singh, 2019). Unlike organic pollutants, heavy metals cannot be biodegraded but can only undergo changes in morphological and valence, migration, and transformation within the environment or food chain (Li et al., 2021). Among the various heavy metals, Cadmium (Cd) is particularly problematic due to its severe and difficult-to-control environmental pollution, making it a critical focus in environmental management research (Rizwan et al., 2016). The traditional treatment technology of cadmium wastewater is mainly achieved by chemical precipitation, ion exchange, evaporation concentration and adsorption (Lee et al., 2024; Liang et al., 2022; Soudani et al., 2022; Arsenie et al., 2022). These methods have the disadvantages of large investment, high consumption cost, complicated processing technology, and secondary pollution.

Microbial remediation technology uses microorganisms as adsorbents, which has the advantages of strong specificity, no secondary pollution, easy operation and low cost (Leong and Chang, 2020; Choudhury and Chatterjee, 2022; Maqsood et al., 2022). However, the study of microbial adsorption of cadmium wastewater is still in small-scale experiment and difficult to be applied in practice. Therefore, it is particularly important to further develop new and efficient biological adsorbents to improve the adsorption capacity of microbial cells for heavy metal ions. The combination of cell surface display and microbial adsorbents has become a powerful strategy for environmental management (Liu et al., 2016). The expression of heterologous metal-binding proteins or peptides on the cell surface through microbial cell surface display systems can specifically bind heavy metals and improve the resistance of microorganisms to metals and cell adsorption capacity. Recently, the combination of microbial metal-binding proteins with surface expression systems has been used to treat heavy metal wastewater, many microorganisms such as *Thiobacillus ferrooxidans*, *Escherichia coli*, and *Pseudomonas* are used as biological adsorbents for wastewater containing cadmium (Su et al., 2022; Li et al., 2021; Meng et al., 2021). Compared with the bacterial system (Valls et al., 2000; Samuelson et al., 2000), the yeast display system has the advantages of protein modification and processing in yeast occurs after translation (Savastru et al., 2022).

In addition to considering how to improve the adsorption of heavy metal ions, another problem is how to adsorb cadmium ions efficiently and selectively from wastewater (Pande et al., 2010). This method enables rapid screening of highly binding to the target and binding to any specific target, including bioactive peptides, proteins, receptors, etc.

However, the application of recombinant bioadsorbents may be problematic due to the complexity of wastewater (Kang et al., 2014). To solve this problem, cell immobilization is a good solution strategy. Because the adsorption of immobilized cells depends on the metal chelation of the peptide, the surface display peptide can still absorb metal ions even if the prepared cells lose their activity. This not only solves the toxicity of complex pollutants to cells, but also facilitates their practical application in factories. At the same time, the immobilized microbial adsorbents can chelate metal ions and accumulate them on the cell surface, and the metal ions can be eluted off without breaking the bacterial cells by only using the eluent, which facilitates the recycling of metal ions and improves the treatment effect of wastewater containing cadmium heavy metals (Juarez Jimenez et al., 2012).

While phage library screening for metal-binding peptides has been widely reported, most studies have focused on commonly occurring heavy metals such as Ni and Zn (Day et al., 2013; Patwardhan et al., 1997; Mooney et al., 2011). There is limited research on the screening of metal-binding peptides for highly toxic metals like Cd^2+^. In this study, *S. cerevisiae* was selected as the research strain, aiming to screen out Cd^2+^ion-binding peptides with strong binding force and high specificity from phage random peptide library, and display these binding peptides on the surface of *S. cerevisiae*, in order to prepare new Cd^2+^ion-binding peptide microbial cell surface adsorbents.

## Materials and methods

### Media and solutions

The following culture media were used for the study: Luria-Bertani (LB) medium, top agar, yeast extract-peptone-dextrose (YPD) medium, MD selective medium [also known as yeast nitrogen base (YNB) medium], and yeast nitrogen base-casamino acids (YNB-CAA) medium.

The following microbial strains were used for the study: *E. coli* ER2738, random phage display library (phage display 12-mer peptide library), *E. coli* 116, and *S. cerevisiae* EBY100.

**Figure 1:**
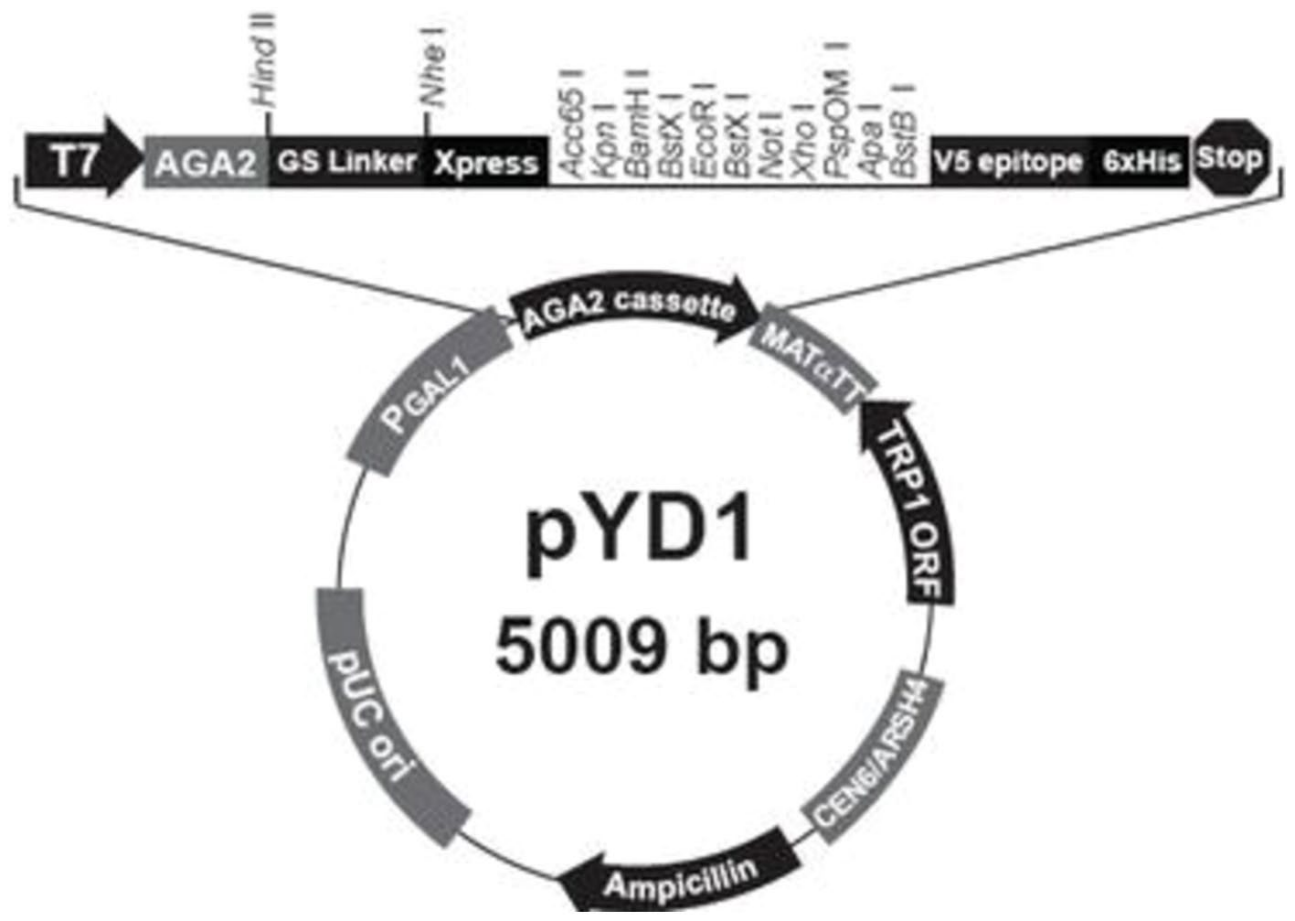
Profile of the pYD1 plasmid.

The pMD-T vector was obtained from Takara for initial cloning of PCR products, and *S. cerevisiae* EBY100 and the pYD1 plasmid were purchased from Beijing Zhongke Quality Inspection Biotechnology Co., Ltd. The primers for phage sequencing were provided by the Phage Randomized Library Kit (New England Biolabs), and additional primers were synthesized by Jinweizhi Biotechnology Co., Ltd (Figure 1; Table 1).

**Table 1:** Primers used in the study.

Name	Sequence 5′ → 3′
-28gIII sequencing primer	HOGTATGGGATTTTGCTAAACAAC
-96gIII sequencing primer	HOCCCTCATAGTTAGCGTACG
pYD1-Forward	AGTAACGTTTGTCAGTAATTGC
pYD1-Reverse	GTCGATTTTGTTACATCTACAC

Restriction endonucleases *Eco*RI and *Xho*I supplied by Fermentas were mainly used for the DNA digestion. RNaseA enzyme (Merck) was used during plasmid extraction. Taq DNA polymerase and Phusion DNA Polymerase were purchased from Shanghai Lifefeng Biotechnology Co., Ltd., used for PCR amplification. Ligase was purchased from Takara.

### Preparation of Cd^2+^ ion chelating resin

Ni-NTA agarose resin (200 μL) was rinsed twice with 500 μL Tris-buffered saline (TBS; 150 mmol/L Tris–HCl [pH 7.2], 150 mmol/L NaCl). Following the washes, 0.5 mol/L EDTA (pH 8.0) was added to the resin. The resin was then washed until its color changed from light blue to colorless. Subsequently, the resin was washed three times with 0.1% TBST (TBS with 0.1% Tween 20). Cd^2+^ were then added to the resin and incubated overnight. The resin was centrifuged and washed three times with TBS, followed by mixing with 0.1% TBST. The prepared Cadmium ion chelating resin (M^+^) was stored at 4°C. For the control (M^−^), the resin was treated with sterilized ultrapure water instead of Cd^2+^.

### Phage display library screening

#### Phage display biopanning

One hundred microliters each of M^+^ and M^−^ resins were placed in separate centrifuge tubes and washed twice with 1,000 μL of 0.1% TBST (pH 7.4). Subsequently, each tube was mixed with 10 μL of the phage display peptide library (2 × 10^11^ PFU/mL) and incubated for 1 h at 25°C. Unbound or loosely bound phages were removed by washing twice with 0.1% TBST. Tightly bound phages were eluted using 500 μL of 20 mmol imidazole; the eluates were washed five times with 0.1% TBST and then eluted twice with 300 μL of 200 mmol imidazole. Next the eluted phages were amplified using *E. coli* ER2738 and purified by a polyethylene glycol precipitation. The phage titer was estimated, and the P/N value (number of M^+^ phage panned/ number of M^−^ phage panned) was calculated. The eluates were amplified, the titers were measured. The same phage titer (2 × 10^11^ PFU/mL) was used for the second round of screening under the same conditions. In the third round of screening, the eluate was washed four times with 0.3% TBST to reduce nonspecific interactions between the target molecule and the recombinant phage. After three rounds of screening, Cd^2+^ ion-binding peptides with high specificity and affinity were obtained. Following elution, the phage was diluted and titrated on *E. coli* ER2738 plates. Phage clones forming a blue plaque were selected for DNA sequencing analysis.

#### Phage tittering

The phages were amplified using the *E. coli* ER2738 strain. The eluted phage was serially diluted into four gradients: 10^−1^ CFU/g, 10^−2^ CFU/g, 10^−3^ CFU/g, and 10^−4^ CFU/g, with three replicates for each dilution. When the cultured *E. coli* ER2738 cells reached mid-logarithmic phase, 200 μL of culture was aliquoted into centrifuge tubes,10 μL of each phage dilution was added and the mixture was rapidly mixed by shaking and incubated at room temperature for 5 min. The phage-infected *E. coli* ER2738 cells were transferred to the upper agar tube preheated at 45°C,swirled briefly then immediately poured onto LB/IPTG/X-gal plates and incubated overnight at 37°C. The number of blue phage plaques was counted to determine phage titer.

Phages were amplified in *E. coli* ER2738 cultured in LB that was supplemented with tetracycline. Ten microliter of phage washing solution was added to 20 mL of ER2738 bacterial solution, and the culture was incubated at 37°C with shaking for 4.5 h. The culture was then transferred to a 50 mL centrifuge tube and centrifuged twice at 10,000 rpm for 10 min at 4°C. The phages in the supernatant were transferred to a clean EP tube and precipitated overnight at 4°C with 20% (w/v) polyethylene glycol-8000 in 2.5 mol/L NaCl (PEG/NaCl). Next the phages were pelleted by centrifugation and resuspended in 1 mL of TBS. Residual bacteria were pelleted by centrifugation (5 min,14,000 rpm),and the supernatant was further precipitated with PEG/NaCl. The obtained phages were then resuspended in 200 μL of TBS and centrifuged to remove the impurities.

#### Amplification and purification of phages

Phages were amplified in *E. coli* ER2738 cultured in LB medium supplemented with tetracycline (20 g L^−1^). After 4.5 h of shaking at 37°C, bacteria were pelleted by centrifugation at 10 min,10,000 rpm. The phages in the supernatant were precipitated overnight at 4°C in 2.5 mol/L PEG-8000-NaCl. The P/N value was calculated using the following equation: P/N = the number of M^+^ phage panned (P)/the number of M^−^ phage panned (N).

#### Plasmids construction and transformation in *S. cerevisiae* EBY100

According to the pYD1 plasmid map, restriction enzymes *Eco*RI and *Xho*I were used for digesting both pYD1 and the four Cd^2+^-binding peptide-encoding genes. The digested products were ligated with DNA ligase and transformed into *E. coli* 116. The positive transformants were verified using PCR and Sanger sequencing. Four Cd^2+^ ion-binding peptide-encoding genes were synthesized artificially either separately or in combination (*gE1*-*gE3*-*gE6*-*gE11*) and inserted into pYD1 plasmid, resulting in the recombinant plasmids pYD1-*gE1*, pYD1-*gE3*, pYD1-*gE6*, and pYD1-*gE11*.

*S. cerevisiae* EBY100 cells were cultured in YPD medium at 30°C until they reached an optical density of 0.6 at 600 nm (OD_600_). The cells were then harvested to prepare competent cells for transformation using the heat-shock method. Briefly, 10 μL of recombinant plasmid DNA, 700 μL of 1× LiAc/10% polyethylene glycol 3350/1× Tris-EDTA buffer, 10 μL of salmon sperm DNA, and *S. cerevisiae* EBY100 competent cells were mixed and incubated at 30°C for 30 min. Subsequently, 88 μL of dimethyl sulfoxide was added, and the mixture was incubated at 42°C for 7 min. Following heat-shock transformation, the cells were plated on YNB (Trp^−^and Leu^+^) medium and incubated at 30°C for 72 h to select positive clones. Confirmation of positive transformants was performed by colony PCR amplification and restriction digestion of plasmid DNA isolated from clones. The recombinant plasmid DNA was retransformed into *E. coli* 116 cells to confirm the correct plasmid construction. Confirmed transformants were grown in YNB-CAA medium containing 20 g L^−1^ glucose at 30°C overnight with shaking. The cells were then transferred to YNB-CAA medium containing 20 g L^−1^ galactose and grown at 25°C with shaking until the OD_600_ reached 0.6. The cells displaying Cd^2+^ ion-binding peptides were harvested, and the binding capacity for Cd^2+^ was calculated as the weight of adsorbed Cd (mg) per gram of yeast sample (g).

#### Immobilization of *S. cerevisiae* cells

The yeast stock stored at −80°C was thawed and plated on a YNB medium plate and then incubated for 36 h. Single colonies were picked and inoculated into YPD medium, followed by overnight incubation at 30°C with shaking at 200 rpm. A 2% inoculum of overnight culture was transferred into 100 mL of fresh YPD medium and grown at 30°C with shaking at 200 rpm until an OD_600_ value of approximately 0.6 was reached. The cells was harvested by transferred centrifuge at 5,000 rpm collected in a 50 mL centrifuge tube. The collected cells were transferred to the YNB-CAA induction medium and incubated overnight at 30°C with shaking at 200 rpm. The overnight-grown cells were then mixed with 1 mL sterile water and combined with a pre-configured sodium alginate solution. The mixture was thoroughly blended and injected into sterile calcium chloride solution using a syringe and fixed at 20°C for 24 h to prepare sodium alginate beads. The immobilized yeast beads were washed three times with sterile water and stored at 4°C until further use. To determine optimal fixation conditions, combinations of different concentrations of sodium alginate and calcium chloride, along with different temperatures were tested. Using of the single-factor experimental design, the concentrations of calcium chloride and sodium alginate, along with a fixed temperature, were selected as test variables. The adsorption ratio was considered as the response variable. An orthogonal experiment with three factors and three levels was conducted to further optimize the immobilization conditions of *S. cerevisiae* (Zarei et al., 2017).

### Statistical analysis

All experiments were performed in triplicate to ensure reproducibility. Data are represented as the mean ± standard deviation (SD). Statistical significance was determined using a *p*-value threshold of <0.05.

## Results

### Screening of Cd^2+^ ion-binding peptides from Ph.D.-12 peptide library

**Figure 2:**
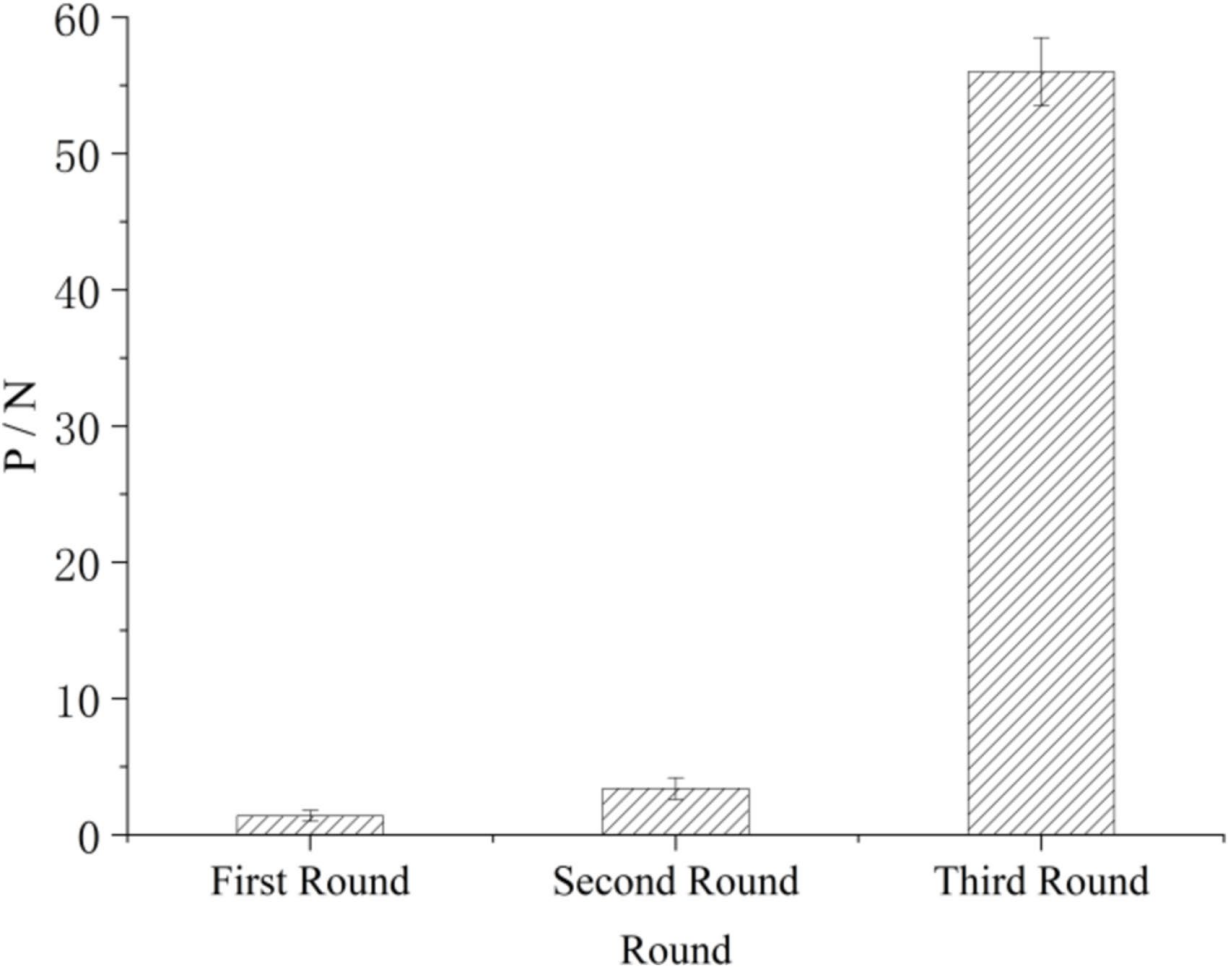
Changes in the P/N values in the three rounds of screening.

A peptide phage library was used to identify hexapeptide exhibiting cadmium binding properties. In the phage panning process, the titer of the phage eluted from M^−^ resin and M^+^ resin was measured in each screening round. When the P/N value reached 50 or more than 60, specific sequence was obtained, and the phage was selected for sequencing. As shown in Figure 2, phage clones were enriched, and specificity was gradually enhanced over the three rounds of screening. The phage screened in the third round exhibited strong specificity, with a P/N value of 56. This finding indicated that the binding peptide obtained had a strong affinity for Cd^2+^ and could specifically bind to it in this round of screening.

The phages obtained in the third round of screening were sequenced, and 12 groups of screened phages were selected and sent for sequencing by Jinweizhi Biotechnology Co., Ltd. After removing incorrect or incomplete sequences, four coding genes for dodecapeptides with a strong Cd^2+^ ion adsorption ability were identified and synthesized by Jinweizhi Biotechnology Co., Ltd. The results are shown in Table 2.

**Table 2:** Results of the sequencing analysis.

Sequence number	Sequence composition
1	ATG *** *** *** *** *** *** *** *** *** *** CGG Met H P N A G H G S L M R
3	GCT *** *** *** *** *** *** *** *** *** *** TCT A D W Y H W R S H S S S
6	GAT *** *** *** *** *** *** *** *** *** *** ACT D Y N Y D R S D S R L T
11	ATG *** *** *** *** *** *** *** *** *** *** GGG M F D G L Y G G E R P G

### Display of Cd^2+^ ion-binding peptides on *S. cerevisiae* EBY100 and Cd^2+^ ion biosorption

In our study, plasmid pYD1 contains genes encoding the peptides *gE1*, *gE3*, *gE*6, and *gE*6 were transformed into *S. cerevisiae* EBY100 (EBY100/pYD1-*gE1*, EBY100/pYD1-*gE3*, EBY100/pYD1-*gE*6, EBY100/pYD1-*gE*6). The original *S. cerevisiae* EBY100 strain was used as a control. EBY100 cannot be grown in cultures lacking tryptophan, while recombinant EBY100 contains pYD1 plasmids that can be grown in cultures lacking tryptophan and the transformants were screened on the cultures lacking tryptophan. After preliminary identification of positive transformants, colony PCR was performed for further verification.

**Figure 3:**
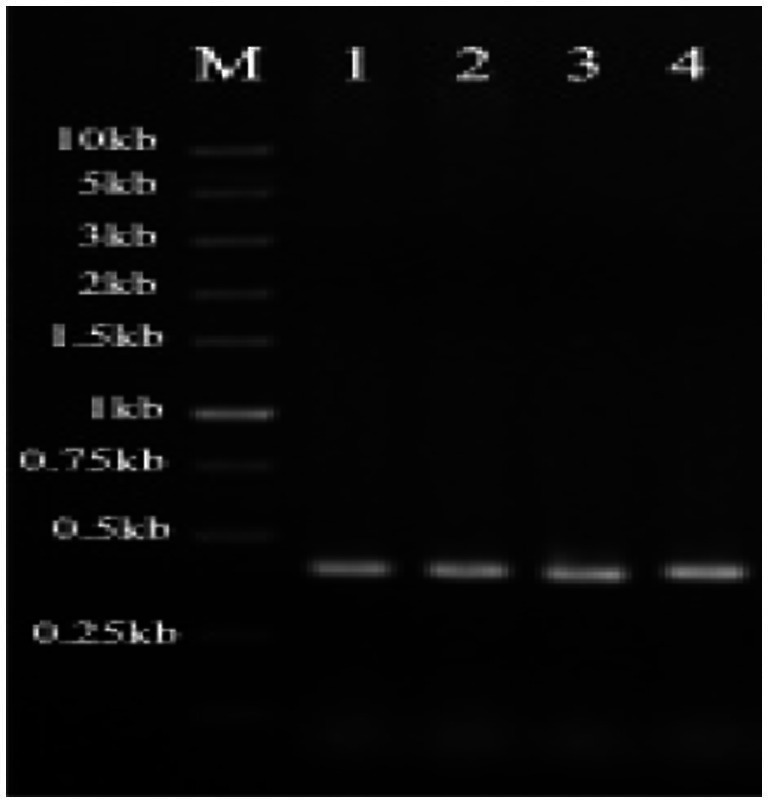
Confirmation of successful transformation of *S. cerevisiae.* M represents DNA Marker, 1 is *gE1* PCR fragment, 2 is *gE3* PCR fragment, 3 is *gE6* PCR fragment, and 4 is *gE11* PCR fragment.

Positive clones were selected for colony PCR validation. The positive clones of *S. cerevisiae* containing plasmids pYD1-*gE1*, pYD1-*gE3*, pYD1-*gE*6, and pYD1-*gE*11 were randomly selected for single-colony PCR, yielding fragments of approximately 0.4 kb (Figure 3). The size of the target band matched the expected size. Because the Cd^2+^ ion-binding peptides are too short to differentiate between empty vectors and recombinant plasmids, gene fragments were sequenced after the colony PCR assay. The results showed that the *gE1*, *gE3*, *gE*6, and *gE*11 inserted correctly into *S. cerevisiae* EBY100.

**Figure 4:**
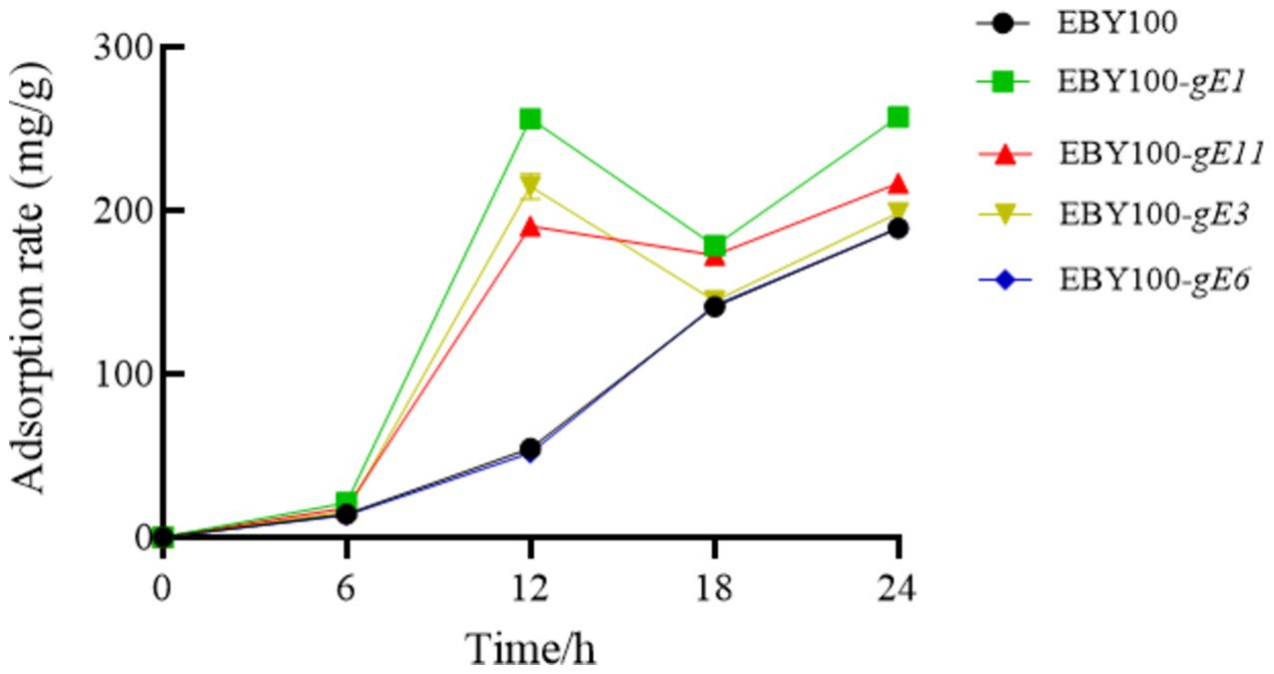
Adsorption rate of EBY100 series strains over time.

*S. cerevisiae* cells were inoculated into YNB medium and incubated with shaking. The cells were then collected, transferred to the induction medium, and cultured until an OD_600_ value of 0.6 was achieved. Subsequently, 0.7 g of Cd was added. The adsorption rate of the EBY100 series strains were determined at 6, 12, 18, and 24 h, and the results were shown in Figure 4. After 24 h of adsorption, the yeast and its recombinant yeast were centrifuged, and the remaining volume of the supernatant, the wet weight of yeast, and the cadmium concentration in the supernatant were determined. Each group was analyzed in triplicate, and the average value was calculated. The recovery rate of each sample was between 95 and 105%. As shown in Table 3, except for *S. cerevisiae* which was transformed with the *gE6* sequence, the adsorption efficiency of other recombinant *S. cerevisiae* strains improved and was 35% higher than that of the control strain.

**Table 3:** Adsorption capacity of recombinant *S. cerevisiae* strains.

Strain	Amount of adsorption (g)	Wet weight of yeast (g)	Adsorption rate (mg/g)	Percentage improvement
EBY100	0.189	1.0	189	contrast
EBY100*-gE1*	0.257	1.0	257	35%(±1%)
EBY100*-gE3*	0.283	1.3	217	14.8%(±0.4%)
EBY100*-gE11*	0.178	0.9	198	4.7%(±0.2%)
EBY100*-gE6*	0.227	1.2	189.2	0.1%(±0.003%)

### Immobilization

Immobilized microbial adsorbents can chelate metal ions on the cell surface, which is convenient for the recycling or sequestration of Cd^2+^ ions, improving the efficacy of heavy metal waste treatment. To determine the optimal yeast immobilization conditions and maximize the sorption of Cd^2+^ ions by yeast cells, based on the data obtained from the single factor study, the effects of three independent variables, including the concentrations of calcium chloride (1.5–2.5%) and sodium alginate (2.5–3.5%), as well as temperature (15–25°C) were investigated using an orthogonal test with three levels to optimize the immobilization of surface-engineered yeast (Table 4).

**Table 4:** Factor/level schedule for the orthogonal experiment.

Factor/level	Calcium chloride concentration (%)	Sodium alginate concentration (%)	Fixed temperature (°C)
1	1.5	2.5	15
2	2.0	3.0	20
3	2.5	3.5	25

As shown in Tables 5, 6, the effect of these three factors on the ability of immobilized yeast cells to adsorb Cd^2+^ ions was that sodium alginate concentration had the greatest effect on adsorb efficiency, followed by calcium chloride concentration, while fixed temperature had the least effect. By using a range analysis, the optimal immobilization conditions for yeast were determined as 2.0% calcium chloride, 3.0% sodium alginate, and a temperature of 25°C. Under these conditions, the adsorption ratio was 68%. The adsorption ratio was determined as the ratio of the adsorbed amount of Cd^2+^ ions by immobilized yeast cells for 24 h to the mass of initially added Cd^2+^ ions.

**Table 5:** Optimization and analysis of conditions for the immobilized *S. cerevisiae* cells.

Treatment number	Calcium chloride concentration (%)	Sodium alginate concentration (%)	Fixed temperature (°C)	Blank column	Adsorption ratio (%)
1	1	1	1	1	62.6667
2	1	2	3	2	66.0000
3	1	3	2	3	60.3333
4	2	1	3	3	63.6667
5	2	2	2	1	68.6667
6	2	3	1	2	62.3333
7	3	1	2	2	61.3333
8	3	2	1	3	62.6667
9	3	3	3	1	58.0000
K1	189.0000	187.6667	187.6667	189.3333	
K2	194.6667	197.3333	190.3333	189.6667	
K3	182.0000	180.6667	187.6667	186.6667	
k1	63.0000	62.5556	62.5556	63.1111	
k2	64.8889	65.7778	63.4444	63.2222	
k3	60.6667	60.2222	62.5556	62.2222	
R	4.2222	5.5556	0.8889	1.0000	
Primary and secondary order	B>A>C				
Excellent level	A2		B2	C2	
Excellent combination	A2B2C2				

**Table 6:** Variance analysis for the optimum immobilization condition.

Factor	Deviation sum of squares	Free degree	Mean square	*F*-value	*p*-value	Significance
Calcium chloride concentration (%)	26.8395	2	13.4198	14.8904	0.0629	*P* < 0.05
Sodium alginate concentration (%)	46.6914	2	23.3457	25.9041	0.0372	
Fixed temperature (°C)	1.5802	2	0.7901	0.8767	0.5328	
Error	1.8025	2	0.9012	1.0000	0.5000	

**Figure 5:**
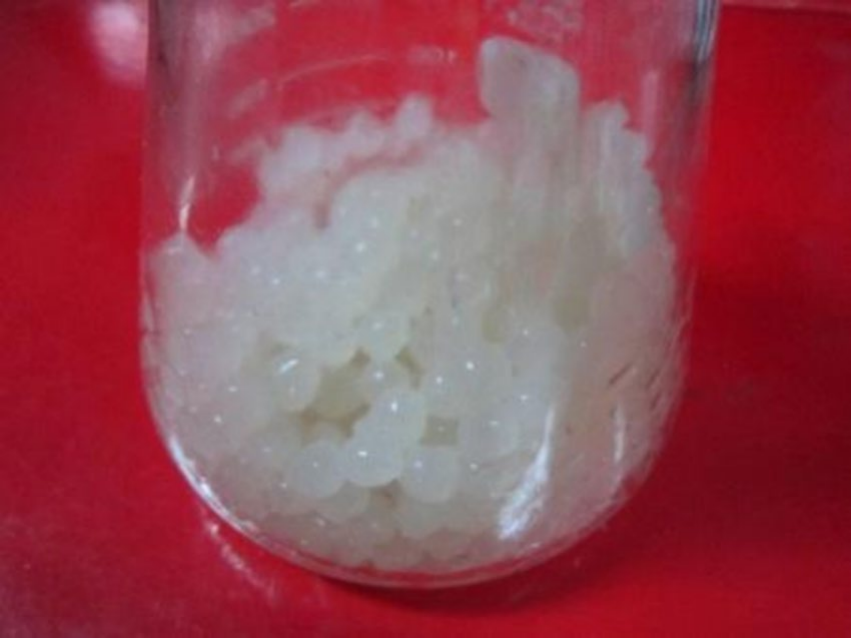
Immobilized cells after Cd^2+^ adsorption.

Five strains of *S. cerevisiae* were immobilized with sodium alginate gels at a mass-to-volume ratio of 1:100. To determine the adsorption effect of the immobilized yeast cells, 0.7 g Cd^2+^ was added to 100 mL medium (Figure 5). As shown in Table 7, except for the EBY100*-gE6* strain, the other strains exhibited good adsorption capacities of up to 55.7%. This finding demonstrated that the immobilized yeast cells had good adsorption performance. The recovery rate of spiking was between 95 and 105% based on calculations for each individual sample.

**Table 7:** Determination of the adsorption capacity of immobilized *S. cerevisiae* cells.

Strain	Adsorption capacity (g)	Wet weight of yeast (g)	Adsorption rate (mg/g)	Percentage increase
EBY100	0.472	64.7	7	Contrast
EBY100*-gE1*	0.439	40.0	10.9	55.7%(±2%)
EBY100*-gE3*	0.39	45.5	8.57	22.4(±0.7%)
EBY100-gE11	0.349	42	8.3	18.5(±0.6%)
EBY100*-gE6*	0.452	63.7	7.09	1.2(±0.04%)

## Discussion

In the present study, the Ph.D.-12 Peptide Library was used to identify Cd^2+^-binding peptides. Three consecutive rounds of biopanning were performed using Cd-NTA agarose as a binding target, with cell surface-binding phages recovered by cell lysis in each round. The P/N value was used to reflect the ability of specific clones to bind Cd^2+^ each round. In the third round of screening, the P/N value reached 56, indicating that the screened peptides had strong specificity for Cd^2+^ ions. By sequencing the selected phages, four usable small peptide sequences were obtained. The analysis showed that histidine was present in both of the sequences. The genes encoding these binding peptides were each expressed on the surface of *S. cerevisiae* EBY100 cells. The results showed that yeast had higher adsorption efficiency for cadmium ion when galactose was induced for 24 h. Compared with the control strain, the adsorption efficiency of the recombinant strain was increased by 35% at most. This finding is consistent with numerous studies showing that histidine is a key component of high-affinity metal-binding peptides, containing an imidazole group that coordinates with metal cations, making it a metal-affinity amino acid (Sharma et al., 2021; Csire et al., 2020).

This result is consistent with the report by Jingshuang et al. (2006) that peptides containing five histidine residues had a stronger affinity for Cd^2+^ than those containing only one histidine. Notably, no cysteine residue was found in the sequences obtained by screening, which may be related to the instability of cysteine in phage screening. Sumiyoshi et al. (2022) developed a peptide-based sequestering agent, AADAAC-(FPGVG)_4_, by introducing the metal-binding sequence AADAAC on the N-terminus of a short ELP, (FPGVG)_4_. In turbidity measurements, AADAAC -(FPGVG)_4_ revealed strong self-assembling ability in the presence of metal ions such as Cd^2+^ and Zn^2+^. The results from colorimetric analysis indicated that AADAAC-(FPGVG)_4_ could capture Cd^2+^ and Zn^2+^. Furthermore, AADAAC-(FPGVG)_4_ that bound to metal ions could be readily recycled by treatment with acidic solution without compromising its metal binding affinity.

Because heavy metals are recalcitrant and do not disintegrate, their immobilization is an ideal remediation strategy (Schommer et al., 2023). Following their immobilization, microbial cells exhibit stronger antitoxic effects and improved capacity for the adsorption of heavy metals (Zhang et al., 2021). Therefore, the study used sodium alginate as a carrier to construct immobilized *S. cerevisiae* cells, the results showed that the adsorption performance of immobilized yeast was the best when the concentration of calcium chloride was 2%, the concentration of sodium alginate was 3%, and the temperature was 25°C. The adsorption efficiency of the recombinant strain was greatly improved by up to 55.7% compared with the control strain in the determination of the adsorption performance of immobilized yeast. This result is comparable to that by Li et al. (2019), who achieved 68.62% adsorption capacity of a recombinant strain for Ni(II) using cell immobilization techniques.

In the process of research, it was also found that this display system has certain limitations in the following aspects. First, there was a lack of options for recombinant yeast, and the yeast growth temperature was strict, below 25°C or above 30°C yeast would stop growing or even die. If the conversion conditions were too harsh, such as the required temperature was too high or too low or the buffering conditions were relatively severe, it may cause the death of yeast, reduce its conversion efficiency, and it was difficult to obtain the target clone. Second, although cadmium ion metal-binding peptide could be displayed on the outside of the cell wall correctly, the display time was timeliness. In the future, better carriers or hosts would be applied to make the metal-binding peptide fixed on the surface of the cell wall and play its role at all times.

## Conclusion

The display of Cd^2+^ ion-binding peptides on yeast creates high-capacity bioadsorbents that are useful to remove Cd from polluted wastewater. Sorption through the cell surface is more powerful than intracellular sorption: (1) sorption through the cell surface enhances the binding of metal compared to the control strain; (2) sorption through the cell surface can wash off the metal ions from the cell surface without breaking the cells, facilitating the recycling of metal ions and yeast cells, which can enhance the treatment efficacy and reduce production costs; (3) sorption through the cell surface is feasible in even dead cells, since non-viable cells can adsorb heavy-metal ions by metabolism-independent surface binding rather than energy-dependent intracellular uptake.

## Data Availability

The original contributions presented in the study are included in the article/Supplementary material, further inquiries can be directed to the corresponding author.
