# Supplementary material for: Enhanced biosorption of cadmium ions on immobilized surface-engineered yeast using cadmium-binding peptides
Source: Front Microbiol. 2024 Nov 15;15:1496843. doi: 10.3389/fmicb.2024.1496843 (PMC11604839; doi:10.3389/fmicb.2024.1496843)
Supplement: Supplementary file 1 [file Supplementary_file_1.docx]

# **Supplementary materials**

**Phage panning process**


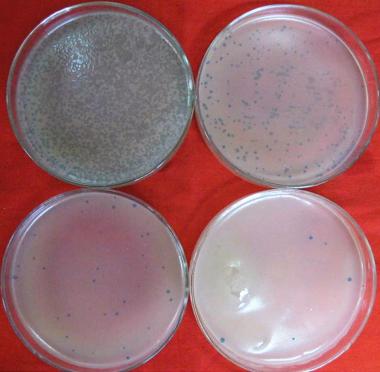

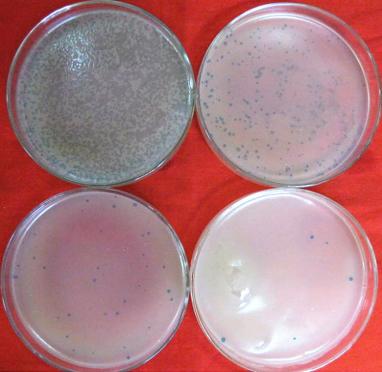

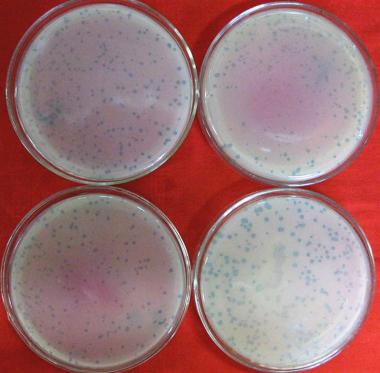


Figure1. Results of the first screening round of amplification.

Note: From left to right, blue phage spots correspond to dilutions of 10^-1^ to 10^-4^ for the eluate after co-incubation of M^-^ resin with phage, dilutions of 10^-1^ to 10^-4^ for the eluate after co-incubation of M^+^ resin with phage, and dilutions of 10^-8^ to 10^-11^ for amplification of the eluate after co-incubation of M^+^ resin with phage.


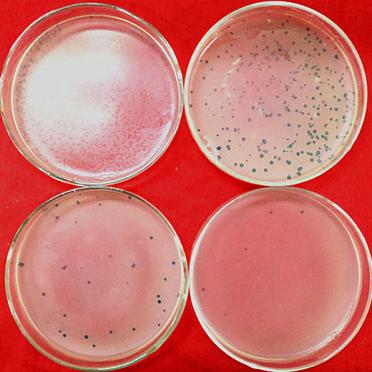

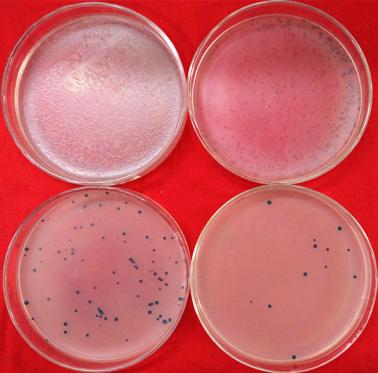

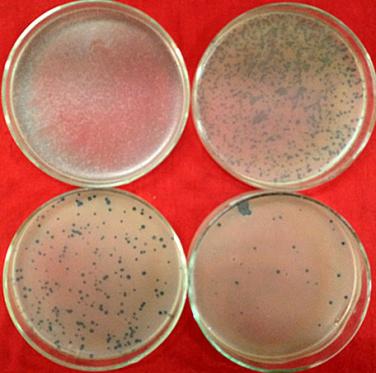


Figure 2. Results of the second screening round of amplification.

Note: From left to right, the blue phage spots correspond to dilutions of 10^-1^ to 10^-4^ for the eluate after co-incubation of M^-^ resin with phage, dilutions of 10^-1^ to 10^-4^ for the eluate after co-incubation of M^+^ resin with phage, and dilutions of 10^-8^ to 10^-11^ for amplification of the eluate after co-incubation of M^+^ resin with phage.


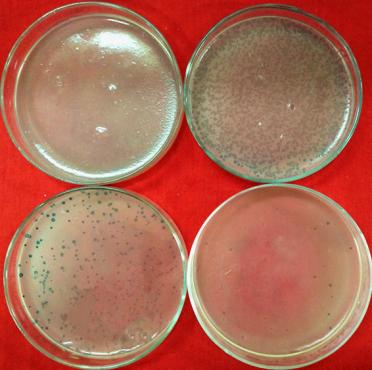

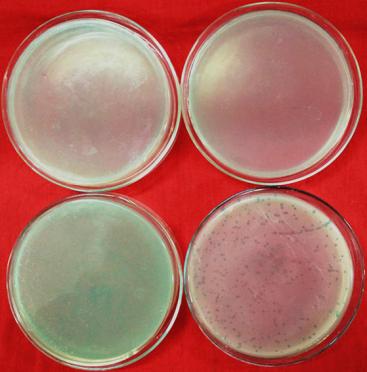


Figure 3. Results of the third screening round of amplification.

Note: From left to right, the blue phage spots obtained in eluates at dilutions of 10^-2^ to 10^-5^ following co-incubation of M^-^ resin with phage and in eluates at dilutions of 10^-2^ to 10^-5^ following co-incubation of M^+^ resin with phage.

As shown in Tables 1–3, phage clones were enriched, and specificity was gradually enhanced over the three rounds of screening.

Table 1. Number of blue phage spots after the first screening round of amplification

| Dilution | M ^-^ blue phage spots from the eluate | M^+^ blue phage spots from the eluate |
| --- | --- | --- |
| 10^-1^ | full plate | full plate |
| 10^-2^ | 576 | 790 |
| 10^-3^ | 75 | 89 |
| 10^-4^ | 6 | 9 |

Table2. Number of blue phage spots after the second screening round of amplification

| Dilution | M ^-^ blue phage spots from the eluate | M^+^ blue spots from the eluate |
| --- | --- | --- |
| 10^-1^ | full plate | full plate |
| 10^-2^ | 385 | 1116 |
| 10^-3^ | 24 | 97.3 |
| 10^-4^ | 3 | 10.7 |

Table 3. Number of blue phage spots after the third screening round of amplification

| Dilution | M ^-^ blue phage spots from the eluate | M^+^ blue phage spots from the eluate |
| --- | --- | --- |
| 10^-4^ | 180 | 全板 |
| 10^-5^ | 13 | 868 |

**Recombinant pYD1 plasmid construction and validation**

The transformants of *E. coli* were cultured overnight, and plasmids were subsequently extracted. The plasmids were digested with *Eco*RI, resulting in bands at approximately 5 kb, indicating the correct plasmid size (Figure 4). The obtained plasmid was subjected to PCR analysis, and the results showed that all fragments were approximately 0.4 kb in size (Figure 5). These results were consistent with the expected size based on primer design. These findings confirm the successful construction of recombinant plasmid pYD1, which was verified by sequencing and comparison with specific short peptide sequences before being transferred into *E. coli* cells.


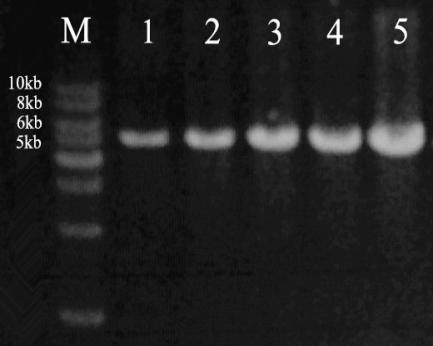


Figure 4. Validation of the recombinant plasmid in E. coli 116 cells by using a single restriction enzyme.

Note: M represents DNA Marker, 1 is pYD1 empty plasmid, 2 is pYD1-gE1 fragment, 3 is pYD1-gE3 fragment, 4 is pYD1-gE6 fragment, and 5 is pYD1-gE11 fragment.


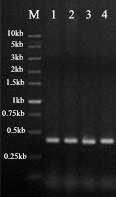


Figure5. Results of PCR analysis for the recombinant plasmid in E. coli 116 cells.

Note: M represents DNA Marker, 1 is gE1 PCR fragment, 2 is gE3 PCR fragment, 3 is gE6 PCR fragment, and 4 is gE11 PCR fragment.

**Determination of cadmium ion standard curve**

A Cd^2+^ standard solution of 10 µg/mL was prepared, from which aliquots of 2.5, 5, 10, 15, 20, 25 mL solutions were taken and diluted to 25 mL, resulting in a series of Cd^2+^ ion concentrations of 1, 2, 4, 6, 8, and 10 µg/mL, respectively. The ionic strength was measured, and a standard curve was plotted (Figure 6). A linear relationship was observed between Cd^2+^ ion concentration and ionic strength within the range of 1 to 10 µg/mL.

Figure 6. Standard curve for determining Cd^2+^ ion concentration.

**Effect of calcium chloride concentration on the adsorption capacity of the immobilized *S. cerevisiae* cells**

The concentration of sodium alginate was fixed at 3%, while the concentration of calcium chloride was varied at 0.5%, 1%, 1.5%, 2%, and 2.5%. The EBY100-gE1 strain was cultured under shaking condition until the OD_600_ value reached approximately 0.6. Following 24-h induction in the induction medium, the yeast cells were collected, and a suspension was prepared using distilled water. Subsequently, 1 mL of yeast suspension was immobilized for the adsorption of Cd^2+^ for 24 h. The adsorption efficiency was measured, and the strength of the particles was determined by the sensory evaluation, with scores of 1, 2, and 3 indicating soft, hard, and slightly hard, respectively. The results demonstrated that within a certain concentration range, higher concentration of Ca^2+^ led to stronger immobilization of yeast cells (Figure 7). The concentration of Ca^2+^ affected the mechanical strength of the immobilized yeast cells; at very high Ca^2+^ concentration, the permeability of the immobilized yeast cells was reduced, thereby affecting their adsorption performance. The optimal immobilization effect was observed at a Ca^2+^ concentration of 2.0%.


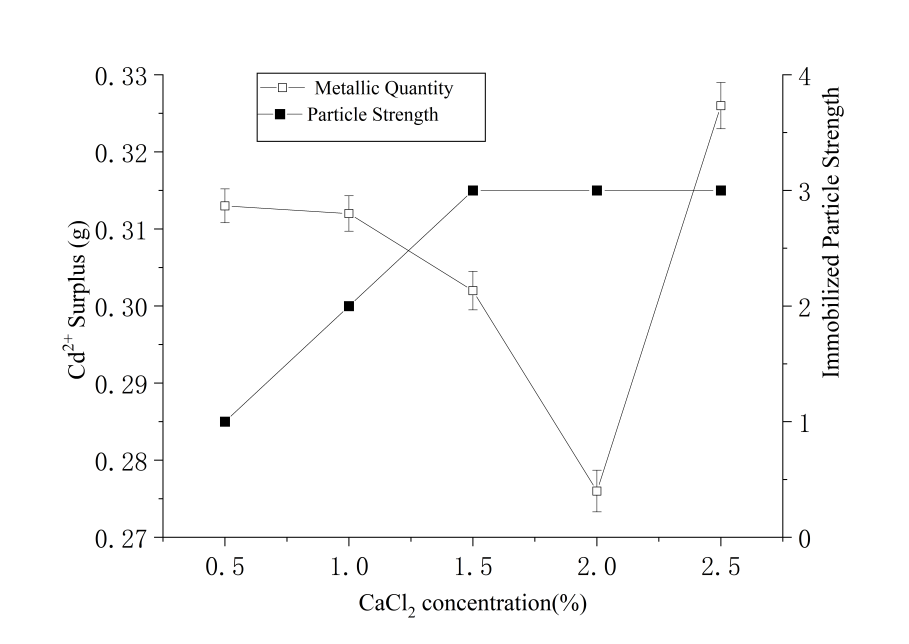


Figure 7. Effect of calcium chloride concentration on the characteristics of the immobilized yeast cells.

**Effect of sodium alginate concentration on the adsorption capacity of the immobilized *S. cerevisiae* cells**

The concentration of calcium chloride was fixed at 2%, while the sodium alginate concentration was varied at 2%, 3%, 4%, and 5%. The *S. cerevisiae* cell suspension was immobilized at 20°C for 24 h. The adsorption efficiency of the immobilized yeast cells was determined according to previously described method, and the strength of the particles was determined by the sensory method, with the scores of 1, 2, and 3 indicating soft, hard, and slightly hard, respectively. The results indicated no significant change in the mechanical strength of the immobilized cells. However, within a certain concentration range, the adsorption efficiency of the immobilized yeast cells gradually decreased with an increase in sodium alginate concentration (Figure 8). This finding suggests that sodium alginate is an important factor in determining the permeability of the immobilized yeast cells. At lower sodium alginate concentration, the relative permeability of the gel was enhanced, which facilitated the adsorption efficiency of yeast cells. Conversely, if the sodium alginate concentration was too low, the network structure of the gel became too large, causing the yeast cells to leak out from the embedding medium and reduce their effectiveness in adsorbing Cd^2+^ ions. The optimal immobilization effect was observed at a sodium alginate concentration of 3%.


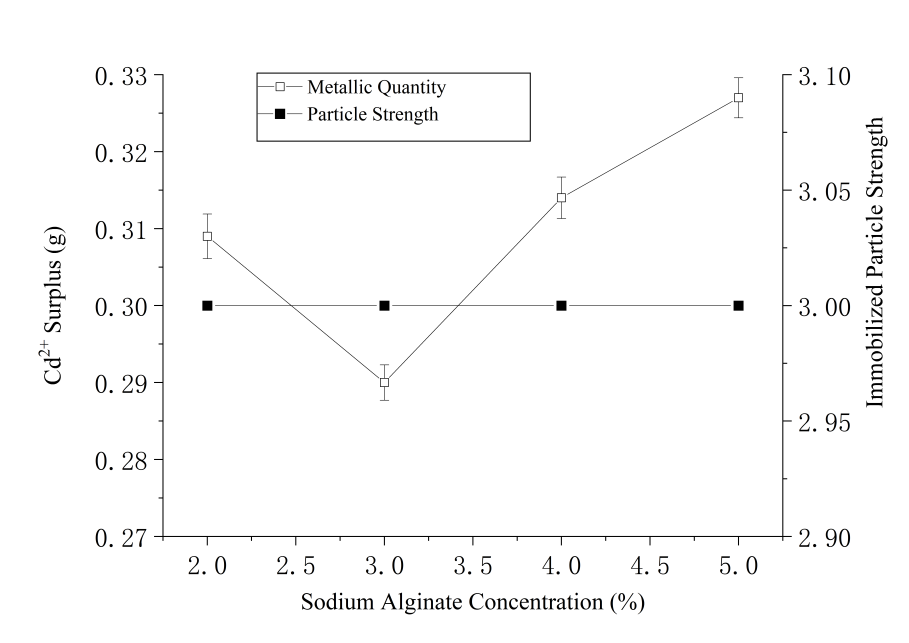


Figure 8. Effect of sodium alginate concentration on the characteristics of immobilized yeast cells.

**Effect of temperature on the adsorption efficiency of the immobilized *S. cerevisiae* cells**

Sodium alginate and calcium chloride concentrations were fixed at 3% and 2%, respectively, and the yeast cell suspension was immobilized at 10℃, 20℃, and 30℃ for 24 h. The adsorption efficiency was determined as previously described. The results showed no significant change in the amount of residual Cd^2+^ ion the solution with varying immobilization temperatures. Moreover, there was no significant difference in the strength of the immobilized yeast particles (Figure 9). Thus, the fixation temperature had no significant effect on the adsorption efficiency of the immobilized yeast cells. For industrial applications, a temperature range of 20℃–25℃ would be more appropriate for immobilization.


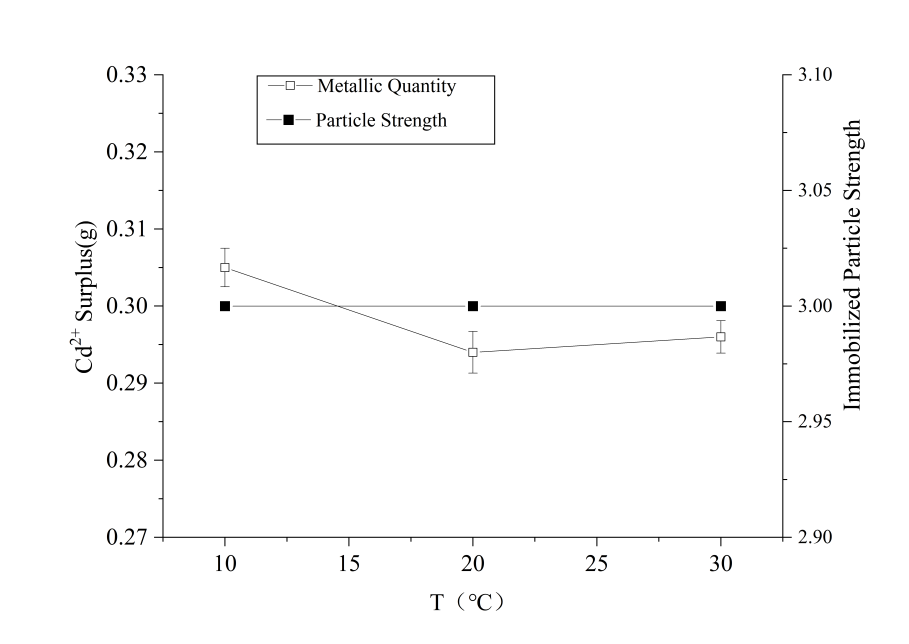


Figure 9. Effect of temperature on the characteristics of the immobilized yeast cells.

**Immobilization**

Five strains of *S. cerevisiae* were immobilized with sodium alginate gels at a mass-to-volume ratio of 1:100. The yeast cells were treated with a sodium alginate carrier, granulated with a 20 mL syringe, and left undisturbed for 24 h (Figure 10). The results suggested that the immobilized *S. cerevisiae* cells obtained by this method yielded immobilized particles of uniform size and shape, with good toughness, resistance to rupture, and a diameter of 3–5 mm, making them easy to recycle.


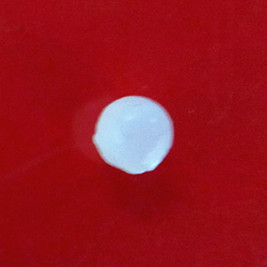


Figure 10. An immobilized cell.
